# Voting-based consensus clustering for combining multiple clusterings of chemical structures

**DOI:** 10.1186/1758-2946-4-37

**Published:** 2012-12-17

**Authors:** Faisal Saeed, Naomie Salim, Ammar Abdo

**Affiliations:** 1 Faculty of Computer Science and Information Systems, University Technology of Malaysia, Johor, Malaysia; 2 Information Technology Department, Sanhan Community College, Sana'a, Yemen; 3 Department of Computer Science, Alhodaida University, Alhodaida, Yemen; 4 LIFL UMR CNRS 8022 Universite′ Lille 1 and INRIA Lille Nord Europe, 59655 Villeneuve d’Ascq cedex, Lille, France

## Abstract

**Background:**

Although many consensus clustering methods have been successfully used for combining multiple classifiers in many areas such as machine learning, applied statistics, pattern recognition and bioinformatics, few consensus clustering methods have been applied for combining multiple clusterings of chemical structures. It is known that any individual clustering method will not always give the best results for all types of applications. So, in this paper, three voting and graph-based consensus clusterings were used for combining multiple clusterings of chemical structures to enhance the ability of separating biologically active molecules from inactive ones in each cluster.

**Results:**

The cumulative voting-based aggregation algorithm (CVAA), cluster-based similarity partitioning algorithm (CSPA) and hyper-graph partitioning algorithm (HGPA) were examined. The F-measure and Quality Partition Index method (QPI) were used to evaluate the clusterings and the results were compared to the Ward’s clustering method. The MDL Drug Data Report (MDDR) dataset was used for experiments and was represented by two 2D fingerprints, ALOGP and ECFP_4. The performance of voting-based consensus clustering method outperformed the Ward’s method using F-measure and QPI method for both ALOGP and ECFP_4 fingerprints, while the graph-based consensus clustering methods outperformed the Ward’s method only for ALOGP using QPI. The Jaccard and Euclidean distance measures were the methods of choice to generate the ensembles, which give the highest values for both criteria.

**Conclusions:**

The results of the experiments show that consensus clustering methods can improve the effectiveness of chemical structures clusterings. The cumulative voting-based aggregation algorithm (CVAA) was the method of choice among consensus clustering methods.

## Background

Chemoinformatics, as defined by Brown [1], is the collection, representation and organisation of chemical data in order to create chemical information, which is applied to create chemical knowledge. It has been used for the process of drug discovery and design, especially in the lead identification and optimisation process, which is known as High-Throughput Screening (HTS).

According to Brown and Martin [2], the advent of high-throughput biological screening methods has given pharmaceutical companies the ability to screen many thousands of compounds in a short time. However, there are many hundreds of thousands of compounds available both in-house and from commercial vendors. Whilst it may be feasible to screen many, or all, of the compounds available, this is undesirable for reasons of cost and time and may be unnecessary if it results in the production of some redundant information. Therefore, there has been a great deal of interest in the use of compound clustering techniques to aid in the selection of a representative subset of all the compounds available [3]. Given a clustering method, which can group structurally similar compounds together, and application of the *similar property principle*[4], which states that structurally similar molecules will exhibit similar physicochemical and biological properties, implies that the selection, or synthesis, and testing of representatives from each cluster produced from a set of compounds should be sufficient to understand the structure-activity relationships of the whole set, without the need to test them all. An appropriate clustering method will, ideally, cluster all similar compounds together whilst separating active and inactive compounds into different sets of clusters [2].

The main objective of clustering is to subdivide data objects into smaller groups known as clusters so that each group exhibits a high degree of intra-cluster similarity and inter-cluster dissimilarity [5]. Many different types of clustering techniques for chemical structures have been used in the literature [6-13].

Brown and Martin [2] considered the Ward’s clustering to be the most efficient method in cluster-based compound selection. However, as it is known, there is no clustering method capable of correctly finding the underlying structure for all data sets. So, the idea of combining different clustering results (consensus clustering) is considered as an alternative approach for improving the quality of the individual clustering algorithms [14].

Consensus clustering involves two main steps: (i) partitions generation and (ii) consensus function. In the first step, many partitions will be generated (the collection of partitions is called ensemble). There are no constraints about how the partitions must be generated. In the partitions generation step, many mechanisms can be applied including the using of: (i) different data representations, (ii) different individual clustering methods, (iii) different parameters initialisation for clustering methods and (iv) data resampling. In the second step, there are two main approaches, i.e. the objects co-occurrence-based and the median partition-based approaches. Voting and graph based consensus clusterings are widely used for the first approach.

Topchy *et al.*[15] and Fred and Jain [16] summarised the main advantages of using consensus clustering in the following terms:

Robustness: The combination process must have better average performance than the single clustering algorithms.

Consistency: The result of the combination should be somehow, very similar to all combined single clustering algorithm results.

Novelty: Cluster ensembles must allow finding solutions unattainable by single clustering algorithms.

Stability: Results with lower sensitivity to noise and outliers.

In chemoinformatics, it is most unlikely that any single method will yield the best classification under all circumstances, even if attention is restricted to a single type of application [17]. Furthermore, the consensus scoring (data fusion) has been successfully used in chemoinformatics and, in particular, for virtual screening [18-25].

Over the last few years, data fusion has become accepted as a simple way of enhancing the performance of existing systems for ligand-based virtual screening by combining the results of two or more screening methods. In some cases, the fused search may even be better than the best individual screening method when averaged over large numbers of searches [20].

Chu *et al.*[17] used consensus clustering methods on sets of chemical compounds represented by 2D fingerprints (ECFP_4), and concluded that consensus methods can outperform the Ward's method, the current standard clustering method for chemoinformatics applications. However, based on the implemented methods, it was not the case if the clustering is restricted to a single consensus method. In this paper, we examined the use of voting and graph-based consensus clustering methods for combining multiple clusterings of chemical structures with different distance measures in order to improve the effectiveness of chemical structures clustering.

## Experimental

### Molecular fingerprints

For the clustering experiments, two molecular fingerprints were developed by Scitegic’s Pipeline Pilot software [26]. The first one was 120-bit ALOGP, which includes octanol-water partitioning coefficient based on Ghose and Crippen’s method [27,28]. ALOGP atom type code is generated based on the molecular hydrophobicity (lipophilicity), usually quantified as log P (the logarithm of 1-octanol/water partition coefficient), which is an important molecular characteristic in drug discovery [28]. The second descriptor was the Scitegic extended-connectivity fingerprints (1024 ECFP_4). The first character E in the fingerprint name denotes the atom abstraction method used to assign initial atom code which is derived from the number of connections to an atom, the element type, the charge and the atomic mass [29].

### Dataset

Experiments were conducted over the most popular chemoinformatics databases: the MDL Drug Data Report database [30]. This database consists of 102516 molecules which was described by Hert *et al.*[21] and used for consensus scoring that combined the results of different similarity searches of a chemical database. Also, it has been used for many virtual screening experiments [31-33]. According to Hert *et al.*[26], the subset dataset was chosen from MDDR database which is quite disparate in nature, some of the molecules being structurally homogeneous (e.g., rennin and HIV-1 protease inhibitors) while others were structurally diverse (e.g., cyclooxygenase and protein kinase C inhibitors); the diversity was estimated by the mean pairwise Tanimoto similarity across each set of active molecules (activity class). The calculations of pairwise Tanimoto similarity were conducted using Pipeline Pilot software. The MDDR dataset contains eleven activity classes (8294 molecules) and the details of this dataset are listed in Table 1. Each row in the table contains an activity class, the number of molecules belonging to the class and the diversity of the class.

**Table 1:** MDDR dataset activity classes

**Activity Index**	**Activity class**	**Active molecules**	**Pairwise similarity**
			**Mean**
31420	Renin Inhibitors	1130	0.290
71523	HIV Protease Inhibitors	750	0.198
37110	Thrombin Inhibitors	803	0.180
31432	Angiotensin II AT1 Antagonists	943	0.229
42731	Substance P Antagonists	1246	0.149
06233	Substance P Antagonists	752	0.140
06245	5HT Reuptake Inhibitors	359	0.122
07701	D2 Antagonists	395	0.138
06235	5HT1A Agonists	827	0.133
78374	Protein Kinase C Inhibitors	453	0.120
78331	Cyclooxygenase Inhibitors	636	0.108

### Ensemble generation

 Every consensus clustering method is made up of two steps: (i) partitions generation and (ii) consensus functions. For the purposes of this paper, the partitions were generated by a single run of multiple individual clustering algorithms (single-linkage, complete linkage, average linkage, weighted average distance, Ward and K-means methods). Every individual clustering used six distance measures in order to generate different ensembles. The thresholds of 500, 600, 700, 800, 900 and 1000 were used to generate partitions with different sizes (number of clusters). The same process was done for each 2D fingerprint in order to study the effectiveness of consensus clusterings on different molecular representations. The distance measures that were used with each clustering technique were Correlation, Cosine, Euclidean, Hamming, Jaccard and Manhattan.

## Methods

### Graph-based consensus clustering

Two graph-based consensus clustering algorithms, proposed by Strehl and Gosh [34], were used to obtain the consensus partition from ensembles generated in the previous step. The two algorithms were developed based on transforming the set of clusterings into a hyper-graph representation. The first algorithm is Cluster-based Similarity Partitioning Algorithm (CSPA) in which a clustering signifies a relationship between objects in the same cluster and can thus be used to establish a measure of pairwise similarity. Because of this similarity measure, CSPA is also categorized under consensus similarity matrix methods. The second algorithm is the Hyper-Graph Partitioning Algorithm (HGPA) in which the cluster ensemble problem is formulated as partitioning the hyper-graph by cutting a minimal number of hyper-edges. Both algorithms were coded by the published cluster ensemble package that is available on (http://www.strehl.com).

For CSPA, the similarity matrix is generated so that each two objects have a similarity of 1 if they are in the same cluster and 0 otherwise. The process is repeated for each clustering method. A *n× n* binary similarity matrix *S* is created where *n* is the total number of objects in the dataset. The entries of *S* are divided by *r,* which is the number of clustering methods. Then, the similarity matrix is used to re-cluster the objects using any reasonable similarity-based clustering algorithm. Here, we view the similarity matrix as graph (vertex = object, edge weight = similarity) and cluster it using graph partitioning algorithm METIS [35] because of its robust and scalable properties in order to obtain the consensus partition.

The HGPA portions the hyper-graph directly. This is done by removing the lower number of hyper-edges. All hyper-edges have the same weight and are searched by cutting the minimum possible number of hyper-edges that partition the hyper-graph in k connected components of approximately the same dimension. For the implementation of this method, the hyper-graphs partitioning package HMETIS [36] was used.

### Voting-based consensus clustering

The cumulative voting-based aggregation algorithm consists of two steps; the first one is to obtain the optimal relabeling for all partitions, which is known as the voting problem. Then, the voting-based aggregation algorithm is used to obtain the aggregated (consensus) partition. The voting-based aggregation algorithm described by Ayed and Kamel [37,38] is modified to be used in this paper.

Let *χ* denote a set of *n* data objects, and let a partition of *χ* into *k* clusters be represented by an *n*×*k* matrix **U** such that ∑ _*q* = 1_^*k*^*ujq* = 1, for ∀ j. Let *u* = {U_*i*_}_*i* = 1_^*b*^ denote an ensemble of partitions. The voting-based aggregation problem is concerned with searching for an optimal relabeling for each partition **V**^***i***^ with respect to representative partition **U**^**0**^ (with *k*^0^ clusters) and for a central aggregated partition denoted as **Ū** that summarises the ensemble partitions. The matrix of coefficients **W**^***i***^, which is a *k*^*i*^ × *k*^0^ matrix of *w*_*lq*_^*i*^ coefficients, is used to obtain the optimal relabeling for ensemble partitions.

In this paper, the fixed-reference approach is used, whereby an initial reference partition is used as a common representative partition for all the ensemble partitions and remains unchanged throughout the aggregation process. Instead of selecting random partition, the partition that is generated by the method, which showed high ability to separate active from inactive molecules in our experiments, is suggested to be the reference partition U^0^; and this method is the Ward’s clustering (the current standard clustering method for Chemoinformatics applications). The cumulative voting-based aggregation algorithm is described as follows:

Cumulative Voting-based Aggregation Algorithm

1. select a partition **U**^***i***^ ∈*u* which is generated by the Ward’s method and assign to **U**^0^

2. for *i*=1 to *b* do

3. W^*i*^ = (U^*i*T^U^*i*^)^− 1^U^*i*^^T^U^0^

4. V^*i*^ = U^*i*^W^*i*^

5. $U^{0}=\frac{i-1}{i}U^{0}+\frac{1}{i}V^{i}$

6. end for

7. **Ū** = **U**^0^.

### Performance evaluation

The results were evaluated based on the effectiveness of the methods to separate active from inactive molecules using two measures: the F-measure [39] and Quality Partition Index (QPI) measure [40]. As defined by [17], if the cluster contains *n* compounds, that *a* of these are active and that there is a total of *A* compounds with the chosen Activity. The precision, *P*, and the recall, *R*, for that cluster are:

(1) $$P=\frac{a}{n}$$

(2) $$R=\frac{a}{A}$$

(3) $$F=\frac{2\mathrm{PR}}{P+R}$$

This calculation is carried out on each cluster and the F-measure is the maximum value across all clusters.

In addition, according to [13], an active cluster can be defined as a non-singleton cluster for which the percentage of active molecules in the cluster is greater than the percentage of active molecules in the dataset as a whole. Let *p* be the number of actives in active clusters, *q* be the number of inactives in active clusters, *r* be the number of actives in inactive clusters (i.e., clusters that are not active clusters) and *s* be the number of singleton actives. The high value occurs when the actives are clustered tightly together and separated from the inactive molecules. The QPI is defined to be:

(4) $$\mathrm{QPI}=\frac{p}{p+q+r+s}$$

## Results and discussion

The ensembles were generated by running the six individual clusterings, each with the six distance measures. Then, the ensembles were combined using voting and graph-based consensus clustering methods: CVAA, CSPA and HGPA. This process was repeated for each fingerprint (ALOGP and ECFP_4).

The mean of F-measure and QPI values were averaged over the eleven activity classes of the dataset. Tables 2, 3, 4 and 5 show the effectiveness of MDDR dataset clustering for ALOGP and ECFP_4 fingerprints. In all tables, the best values for F-measure and QPI of consensus clustering methods for each column were bold-faced for ease of reference.

**Table 2:** Effectiveness of clustering of MDDR dataset using F-Measure: ALOGP Fingerprint

**Clustering method**			**No. of clusters**					
			**500**	**600**	**700**	**800**	**900**	**1000**
Consensus clustering	CVAA	Correlation	26.80	21.96	18.96	18.49	17.6	15.45
		Cosine	24.79	21.72	19.01	18.19	16.46	14.81
		Euclidean	**27.96**	**23.75**	**22.68**	**24.30**	**21.17**	**19.95**
		Hamming	24.02	20.48	16.31	16.85	14.95	14.68
		Jaccard	23.58	21.96	18.01	18.46	16.72	15.35
		Manhattan	27.03	25.23	21.16	20.36	19.10	19.05
	CSPA	Correlation	5.06	4.65	4.16	3.56	3.35	3.04
		Cosine	5.17	4.65	4.08	3.62	3.37	3.05
		Euclidean	5.12	4.64	4.04	3.61	3.38	3.00
		Hamming	5.30	4.74	4.16	3.62	3.54	3.13
		Jaccard	5.31	4.82	4.15	3.77	3.48	3.13
		Manhattan	5.33	4.80	4.21	3.62	3.45	3.05
	HGPA	Correlation	7.13	5.48	5.45	4.65	4.35	4.37
		Cosine	8.06	6.04	5.03	4.52	4.45	4.08
		Euclidean	7.08	6.55	5.65	4.67	4.56	4.60
		Hamming	8.37	5.73	4.94	5.29	4.97	4.93
		Jaccard	7.63	6.22	5.98	4.53	5.24	3.92
		Manhattan	7.72	6.48	5.23	5.35	4.90	4.12
Individual clustering	Ward's method		9.93	9.19	8.19	7.17	6.67	6.44

**Table 3:** Effectiveness of clustering of MDDR dataset using F-Measure: ECFP_4 Fingerprint

**Clustering method**			**No. of clusters**					
			**500**	**600**	**700**	**800**	**900**	**1000**
Consensus clustering	CVAA	Correlation	33.58	29.81	24.44	20.09	18.41	17.43
		Cosine	34.75	31.32	24.97	20.26	18.46	17.73
		Euclidean	25.43	23.34	20.51	19.13	16.47	14.64
		Hamming	25.48	24.04	20.23	19.62	17.31	14.73
		Jaccard	**35.71**	**33.17**	**28.66**	**21.8**	**19.63**	**18.86**
		Manhattan	25.41	23.98	20.30	19.53	17.25	14.65
	CSPA	Correlation	5.53	4.88	4.23	3.85	3.6	3.18
		Cosine	5.43	4.88	4.28	3.91	3.55	3.10
		Euclidean	5.47	4.87	4.17	3.79	3.53	3.33
		Hamming	5.45	4.82	4.23	3.87	3.58	3.19
		Jaccard	5.51	4.99	4.25	3.99	3.62	3.20
		Manhattan	5.44	4.85	4.23	3.89	3.62	3.20
	HGPA	Correlation	7.01	6.2	5.21	4.5	4.16	3.68
		Cosine	6.83	5.95	5.29	4.47	4.21	3.93
		Euclidean	7.29	5.82	5.29	4.39	4.48	3.94
		Hamming	7.01	5.83	5.29	4.50	4.37	3.69
		Jaccard	6.87	5.91	5.31	4.81	4.80	3.66
		Manhattan	7.81	5.17	5.38	4.61	4.66	3.68
Individual clustering	Ward's method		11.61	10.71	9.04	8.29	7.64	7.02

**Table 4:** Effectiveness of clustering of MDDR dataset using QPI: ALOGP Fingerprint

**Clustering method**			**No. of clusters**					
			**500**	**600**	**700**	**800**	**900**	**1000**
Consensus clustering	CVAA	Correlation	43.84	47.38	48.72	50.70	53.41	54.06
		Cosine	45.60	46.08	47.56	50.46	53.79	54.50
		Euclidean	44.43	45.54	47.95	48.65	52.68	54.86
		Hamming	53.13	56.08	59.07	60.58	64.02	67.76
		Jaccard	**57.86**	**60.62**	**64.07**	**66.49**	**70.68**	**73.53**
		Manhattan	56.01	58.10	60.99	61.86	64.56	65.97
	CSPA	Correlation	46.81	50.04	51.72	51.78	54.23	56.36
		Cosine	46.04	49.49	51.42	52.11	54.48	55.92
		Euclidean	46.20	49.86	51.05	51.88	54.36	56.33
		Hamming	54.67	58.50	60.27	61.78	62.33	65.66
		Jaccard	55.03	59.13	60.84	61.03	63.73	67.44
		Manhattan	55.08	59.00	59.10	60.84	61.78	64.61
	HGPA	Correlation	47.59	49.51	52.39	54.45	56.86	58.56
		Cosine	45.58	48.44	52.78	54.42	56.36	58.70
		Euclidean	46.92	51.41	53.20	54.75	57.00	58.97
		Hamming	55.24	58.48	60.30	63.99	68.21	69.22
		Jaccard	55.71	59.89	64.10	65.15	70.48	71.60
		Manhattan	54.84	58.98	62.73	63.58	65.85	69.97
Individual clustering	Ward's method		52.33	54.86	56.90	59.00	61.33	63.17

**Table 5:** Effectiveness of clustering of MDDR dataset using QPI: ECFP_4 Fingerprint

**Clustering method**			**No. of clusters**					
			**500**	**600**	**700**	**800**	**900**	**1000**
Consensus clustering	CVAA	Correlation	74.86	78.02	82.39	84.16	85.71	87.04
		Cosine	74.79	78.12	81.85	84.78	85.91	87.18
		Euclidean	71.04	74.92	78.41	81.91	84.47	86.80
		Hamming	70.99	74.36	78.47	81.68	84.24	86.28
		Jaccard	**83.48**	**87.01**	**88.72**	**90.98**	**90.67**	**92.05**
		Manhattan	70.74	74.26	78.52	81.74	84.12	86.09
	CSPA	Correlation	70.58	73.29	74.86	76.86	79.17	82.03
		Cosine	71.23	71.85	76.43	76.55	78.06	81.21
		Euclidean	65.33	67.09	72.49	72.73	74.50	78.75
		Hamming	64.68	66.82	69.88	71.25	74.17	76.64
		Jaccard	69.91	71.73	74.20	76.01	77.72	79.26
		Manhattan	63.07	65.77	68.83	71.50	74.06	77.33
	HGPA	Correlation	72.61	74.85	76.4	78.32	80.22	82.26
		Cosine	72.06	74.25	77.21	79.54	81.02	83.31
		Euclidean	70.71	72.82	75.02	76.80	80.50	82.66
		Hamming	69.45	72.21	74.08	77.71	79.67	82.36
		Jaccard	67.88	70.58	73.93	76.56	77.65	79.67
		Manhattan	72.74	72.14	75.68	77.94	81.42	82.97
Individual clustering	Ward's method		75.83	79.88	83.34	84.25	86.49	88.25

Visual inspection of F-measure and QPI values in Tables 2, 3, 4 and 5 enables comparisons to be made between the effectiveness of three consensus clustering methods and the Ward’s method. In addition, two fingerprints were used for the experiments in order to study the effectiveness of consensus clustering on different representations of molecular dataset.

For clustering of MDDR dataset which is represented by ALOGP fingerprint (Tables 2 and 4), the performance of voting-based consensus method (CVAA) outperformed the Ward’s method and the graph-based consensus methods (CSPA and HGPA) using the two criteria: F-measure and QPI. The highest F-measure values were obtained by using Euclidean distance measure with individual clustering methods in the ensemble generation step. While, using the QPI measure, the highest values were obtained by using the Jaccard distance measure. Moreover, the ensembles generated by the other distance measures showed a better performance of CVAA than Ward and graph-based consensus clustering methods using both criteria.

Similarly, the results in Tables 3 and 5 show that, when ECFP_4 fingerprint is used, the CVAA consensus clustering performed very well and outperformed Ward and graph-based consensus clustering methods using F and QPI measures. The Jaccard distance measure was the method of choice to generate the ensembles, which gives the highest values for both criteria.

Some statistical significance tests (T-tests) were performed to show the improvements achieved by the consensus clustering methods, as shown in Tables 6 and 7. It was found that the performance of voting-based consensus method is statistically significant when using both criteria.

**Table 6:** ***T*-test statistical significance testing using F-measure**

	**Paired differences**					**Sig. (2-tailed)**
	**Mean**	**Std. deviation**	**Std. error mean**	**95% Confidence interval of the difference**		
				**Lower**	**Upper**	
a) ALOGP:						
Pair 1: CVAA - Wards	15.37	1.77	0.72	13.50	17.23	0.000004
Pair 2: CVAA -CSPA	19.16	2.11	0.86	16.95	21.38	0.000003
Pair 3: CVAA - HGPA	17.24	1.75	0.71	15.40	19.08	0.000002
b) ECFP_4:						
Pair 1: CVAA - Ward	17.25	5.48	2.24	11.49	23.01	0.000589
Pair 2: CVAA - CSPA	22.01	6.41	2.62	15.27	28.75	0.000391
Pair 3: CVAA – HGPA	20.84	5.95	2.43	14.58	27.09	0.000357

**Table 7:** T-test statistical significance testing using QPI measure

	**Paired differences**					**Sig. (2-tailed)**
	**Mean**	**Std. deviation**	**Std. error mean**	**95% Confidence interval of the difference**		
				**Lower**	**Upper**	
a) ALOGP:						
Pair 1: CVAA - Wards	7.61	1.92	0.78	5.58	9.63	0.000199
Pair 2: CVAA -CSPA	4.20	2.08	0.85	2.02	6.39	0.004290
Pair 3: CVAA - HGPA	1.62	1.06	0.43	0.49	2.74	0.013842
b) ECFP_4:						
Pair 1: CVAA - Ward	5.81	1.60	0.65	4.12	7.49	0.000301
Pair 2: CVAA - CSPA	12.31	1.49	0.61	10.74	13.88	0.000005
Pair 3: CVAA – HGPA	10.77	1.50	0.61	9.20	12.34	0.000010

Tables 6 and 7 display a number of parameters: mean value, standard deviation, standard error and significance values for the pairs of the best F-measure and QPI values of clustering methods which are ((CVAA, Ward’s method), (CVAA, CSPA) and (CVAA, HGPA)) compared in the paired samples t-test procedure. The paired-samples t-test procedure compares the means of two variables that represent the same group at different cluster size. Since the paired samples t-test compares the means for the two variables, it is quite useful to know what the mean values are. A low significance value for the t-test (typically less than 0.05) indicates that there is a significant difference that was satisfied between two variables. In Tables 6 and 7, it was noted that the significance field (Sig. (2-tailed)) in terms of F-measure for AlogP is: CVAA-Ward’s method (0.000004), CVAA-CSPA (0.000003) and CVAA-HGPA (0.000002), and for ECFP_4: CVAA-Ward’s method (0.000589), CVAA-CSPA (0.000391) and CVAA-HGPA (0.000357). Similarly, the significance field (Sig. (2-tailed)) in terms of QPI values for AlogP is: CVAA-Ward’s method (0.000199), CVAA-CSPA (0.004290) and CVAA-HGPA (0.013842), and for ECFP_4 is: CVAA -Ward’s method (0.000301), CVAA-CSPA (0.000005) and CVAA-HGPA (0.000010). In addition, the significance value is low in F-measure and QPI values and the confidence interval for the mean difference does not contain zero. It is then concluded that the consensus clustering method, CVAA, obtained significant results by using F-measure and QPI values compared to Ward and graph-based consensus clustering methods. Moreover, the CVAA method is more efficient because the computational complexity of CVAA is O (n) which is better than other consensus clustering methods such as CSPA that has complexity of O (n^2^), where n is the number of data objects [14].

## Conclusions

The experiments results show that consensus clustering methods can improve the effectiveness of chemical structures clusterings. The cumulative voting-based aggregation algorithm CVAA was the method of choice among consensus clustering methods. The performance of CVAA consensus clustering significantly outperforms Ward and graph-based consensus clustering methods (CSPA and HGPA) using F and QPI measures for both ALOGP and ECFP_4 fingerprints, while the graph-based consensus methods outperform the Ward’s method only for ALOGP using QPI measure. The experiments reported here suggest that voting-based consensus clustering can perform very well when the partitions are generated by a single run of multiple individual clusterings that use Jaccard and Euclidean distance measures in the ensemble generation process.

## Competing interests

The authors declare that they have no competing interests.

## Authors’ contributions

FS is a PhD candidate and performed the experiments under the supervision of NS and AA. All authors read and approved the final manuscript.

## References

[B1] Brown FK Chemoinformatics: what is it and how does it impact drug discovery Annu Rep Med Chem 1998 33 375 384

[B2] Brown RD Martin YC Use of structure-activity data to compare structure-based clustering methods and descriptors for use in compound selection J Chem Inf Comput Sci 1996 36 572 584 10.1021/ci9501047

[B3] Willett P Winterman V Bawden D Implementation of non-hierarchic cluster analysis methods in chemical information systems: selection of compounds for biological testing and substructure search output J Chem Inf Comput Sci 1986 26 109 118 10.1021/ci00051a005

[B4] Johnson M Maggiora GM Concepts and Applications of Molecular Similarity 1990 New York: Wiley

[B5] Everitt BS Landau S Leese M Cluster Analysis 2001 4 London: Edward Arnold

[B6] Adamson GW Bush JA A method for the automatic classification of chemical structures Information Storage and Retrieval 1973 9 561 568 10.1016/0020-0271(73)90059-4

[B7] Downs GM Barnard JM Clustering of chemical structures on the basis of two-dimensional similarity measures J Chem Inf Comput Sci 1992 32 644 649 10.1021/ci00010a010

[B8] Willett P Similarity and Clustering in Chemical Information Systems 1987 Letchworth: Research Studies Press

[B9] Downs GM Willett P Fisanick W Similarity searching and clustering of chemical-structure databases using molecular property data J Chem Inf Comput Sci 1994 34 1094 1102 10.1021/ci00021a011

[B10] Brown RD Martin YC The information content of 2D and 3D structural descriptors relevant to ligand–receptor binding J Chem Inf Comput Sci 1997 37 1 9 10.1021/ci960373c

[B11] Schuffenhauer A Brown N Ertl P Jenkins JL Selzer P Hamon J Clustering and rule-based classifications of chemical structures evaluated in the biological activity space J Chem Inf Model 2007 47 2 325 336 10.1021/ci6004004 17286395

[B12] Holliday JD Rodgers SL Willet P Clustering files of chemical structures using the fuzzy k-means clustering method J Chem Inf Comput Sci 2004 44 894 902 10.1021/ci0342674 15154754

[B13] Varin T Bureau R Mueller C Willett P Clustering files of chemical structures using the Székely–Rizzo generalization of Ward's method J Mol Graph Model 2009 28 2 187 195 10.1016/j.jmgm.2009.06.006 19640752

[B14] Vega-Pons S Ruiz-Schulcloper J A survey of clustering ensemble algorithms Int J Pattern Recognit Artificial Intelligence 2011 25 Issue 3 337 372

[B15] Topchy A Jain AK Punch W A mixture model of clustering ensembles Proc. SIAM Intl. Conf. on Data Mining 2004

[B16] Fred ALN Jain AK Combining multiple clustering using evidence accumulation IEEE Trans Patt Anal Mach Intell 2005 27 835 850 10.1109/TPAMI.2005.113 15943417

[B17] Chu C-W Holliday J Willett P Combining multiple classifications of chemical structures using consensus clustering Bioorgan Med Chem 2012 20 18 5366 5371 10.1016/j.bmc.2012.03.010 22484008

[B18] Feher M Consensus scoring for protein-ligand interactions Drug Discov Today 2006 11 421 428 10.1016/j.drudis.2006.03.009 16635804

[B19] Salim N Holliday JD Willett P Combination of fingerprint-based similarity coefficients using data FusionJ Chem Inf Comput Sci 2003 43 435 442 10.1021/ci025596j 12653506

[B20] Willet P Enhancing the effectiveness of ligand-based virtual screening using data fusion QSAR Comb Sci 2006 25 1143 1152 10.1002/qsar.200610084

[B21] Hert J Willett P Wilton DJ Acklin P Azzaoui K Jacoby E Schuffenhauer A New methods for ligand-based virtualscreening: use of data fusion and machinelearning to enhancethe effectiveness of similarity searching J Chem Inf Model 2006 46 462 470 10.1021/ci050348j 16562973

[B22] Whittle M Gillet VJ Willett P Analysis of data fusion methods in virtual screening: Similarity and group fusion J Chem Inf Model 2006 6 2206 2219 17125165 10.1021/ci0496144

[B23] Chen B Mueller C Willett P Combination rules for GroupFusion in similarity-based virtual screening MolInf 2010 29 533 541 10.1002/minf.201000050 27463331

[B24] Rivera-Borroto OM Marrero-Ponce Y García de la Vega JM Grau-Ábalo RC Comparison of combinatorial clustering methods on pharmacological data sets represented by machine learning-selected real molecular descriptors J Chem Inf Model 2011 51 12 3036 3049 10.1021/ci2000083 22098113

[B25] Svensson F Karlen A Skold C Virtual Screening DataFusion Using Both Structure- and Ligand-Based Methods 2011 Model: J. Chem. Inf 10.1021/ci2004835 22148635

[B26] Pipeline Pilot software SciTegic Accelrys Inc 2008 San Diego: Accelrys Inc website http://www.accelrys.com/

[B27] Ghose AK Crippen GM Atomic physicochemical parameters for three-dimensional structure-directed quantitative structure-activity relationships 1. Partition coefficients as a Measure of hydrophobicity J Comput Chem 1986 7 565 577 10.1002/jcc.540070419 3558506

[B28] Ghose AK Viswanadhan VN Wendoloski JJ Prediction of hydrophobic (lipophilic) properties of small organic molecules using fragmental methods: An analysis of ALOGP and CLOGP methods J Phys Chem A 1998 102 3762 3772 10.1021/jp980230o

[B29] Chen L Li Y Zaho Q Peng H Hou T ADME evaluation in drug discovery. 10. Predictions of Pglycoprotein inhibitors using recursive partitioning and naive Bayesian classification techniques Mol Pharm 2011 8 889 900 10.1021/mp100465q 21413792

[B30] Sci Tegic Accelrys Inc., the MDL Drug Data Report (MDDR) database is available from at http://www.accelrys.com/ (accessed 1st of November 2012)

[B31] Abdo A Chen B Mueller C Salim N Willett P Ligand-based virtual screening using bayesian networks J Chem Inf Model 2010 50 1012 1020 10.1021/ci100090p 20504032

[B32] Abdo A Salim N New fragment weighting scheme for the bayesian inference network in ligand-based virtual screening J Chem Inf Model 2011 51 25 32 10.1021/ci100232h 21155550

[B33] Abdo A Saeed F Hentabli H Ali A Salim N Ahmed A Ligand expansion in ligand-based virtual screening using relevance feedback J Comput-Aided Mol Des 2012 26 279 287 10.1007/s10822-012-9543-4 22249773

[B34] Strehl A Ghosh J Cluster ensembles—a knowledge reuse framework for combining multiple partitions J Machine Learning Res 2002 3 583 617

[B35] Karypis G Kumar V A fast and high quality multilevel scheme for partitioning irregular graphs SIAM J Scient Comput 1998 20 359 392 10.1137/S1064827595287997

[B36] Karypis G Aggarwal R Kumar V Shekhar S Multilevel hypergraph partitioning: Application in VLSI domain Proceedings of the 34th annual Design Automation Conference 1997 526 529 ACM

[B37] Ayad HG Kamel MS Cumulative voting consensus method for partitions with a variable number of clusters IEEE Trans Pattern Anal Mach Intell 2008 30 1 160 173 January 18000332 10.1109/TPAMI.2007.1138

[B38] Ayad HG Kamel MS On voting-based consensus of cluster ensembles Patt Recogn 2010 43 1943 1953 10.1016/j.patcog.2009.11.012

[B39] Van Rijsbergen CJ Information Retrieval 1979 2 London: Butterworths

[B40] Varin T Saettel N Villain J Lesnard A Dauphin F Bureau R Rault SJ 3D Pharmacophore, hierarchical methods, and 5-HT4 receptor binding data Enzyme Inhib Med Chem 2008 23 593 603 10.1080/14756360802204748 18821249

